# Asymptomatic *Clostridium difficile* Colonisation and Onward Transmission

**DOI:** 10.1371/journal.pone.0078445

**Published:** 2013-11-12

**Authors:** David W. Eyre, David Griffiths, Alison Vaughan, Tanya Golubchik, Milind Acharya, Lily O’Connor, Derrick W. Crook, A. Sarah Walker, Tim E. A. Peto

**Affiliations:** 1 National Institute for Health Research Oxford Biomedical Research Centre, University of Oxford, Oxford, Oxfordshire, United Kingdom; 2 Oxford University Hospitals NHS Trust, John Radcliffe Hospital, Oxford, Oxfordshire, United Kingdom; Cornell University, United States of America

## Abstract

**Introduction:**

Combined genotyping/whole genome sequencing and epidemiological data suggest that in endemic settings only a minority of *Clostridium difficile* infection, CDI, is acquired from other cases. Asymptomatic patients are a potential source for many unexplained cases.

**Methods:**

We prospectively screened a cohort of medical inpatients in a UK teaching hospital for asymptomatic *C. difficile* carriage using stool culture. Electronic and questionnaire data were used to determine risk factors for asymptomatic carriage by logistic regression. Carriage isolates were compared with all hospital/community CDI cases from the same geographic region, from 12 months before the study to 3 months after, using whole genome sequencing and hospital admission data, assessing particularly for evidence of onward transmission from asymptomatic cases.

**Results:**

Of 227 participants recruited, 132 provided ≥1 stool samples for testing. 18 participants were culture-positive for *C. difficile*, 14/132(11%) on their first sample. Independent risk factors for asymptomatic carriage were patient reported loose/frequent stool (but not meeting CDI criteria of ≥3 unformed stools in 24 hours), previous overnight hospital stay within 6 months, and steroid/immunosuppressant medication in the last 6 months (all p≤0.02). Surprisingly antibiotic exposure in the last 6 months was independently associated with decreased risk of carriage (p = 0.005). The same risk factors were identified excluding participants reporting frequent/loose stool. 13/18(72%) asymptomatically colonised patients carried toxigenic strains from common disease-causing lineages found in cases. Several plausible transmission events to asymptomatic carriers were identified, but in this relatively small study no clear evidence of onward transmission from an asymptomatic case was seen.

**Conclusions:**

Transmission events from any one asymptomatic carrier are likely to be relatively rare, but as asymptomatic carriage is common, it may still be an important source of CDI, which could be quantified in larger studies. Risk factors established for asymptomatic carriage may help identify patients for inclusion in such studies.

## Introduction

Traditionally most *Clostridium difficile* infection (CDI) cases have been thought to result from healthcare-based exposure to other cases [1] and this forms the basis for many prevention measures [2]. However, combined genotyping and hospital admission data from two European cohorts suggest that, in these endemic settings with appropriate infection control, contact with symptomatic cases only accounts for the minority of disease [3], [4]. Applying the greater discriminatory power of bacterial whole genome sequencing in one of these cohorts demonstrates the majority of cases over the 3 year study period are sufficiently genetically diverse that they cannot be related by direct or indirect transmission irrespective of the route of transmission [5].

Data from previous studies suggest *C. difficile* is typically acquired shortly before onset of disease [6], with long term carriage protective against CDI [7], [8]. Therefore, although *C. difficile* is widely found in the environment [9], recent exposure to asymptomatic individuals, particularly in a healthcare setting, represents a compelling potential source for many currently unexplained CDI cases and a potential area for intervention. Between 4% and 15% of healthy adults may be asymptomatically colonised [10], [11]. In hospitalised adults, asymptomatic carriage rates reported in a large Canadian study were 184/4143 (4.4%) at admission with a further 123 (3.0%) becoming asymptomatically colonized during their hospital stay. However the wards included were selected to be a mix of previously high and low CDI incidence wards, and therefore, despite the considerable size of the study, generalising the findings is difficult [6]. Five earlier studies, with a total of 1755 patients across a range of specialties, report asymptomatic carriage rates at admission of 6–11%, and acquisition rates of 4–21%, with more than 63% of these patients remaining asymptomatic [12]. Colonisation in young children is common, with rates ∼35% in the first year of life falling to ∼15% by 1–8 years [13]. High colonisation rates are also described in long-term care facilities, 51% in the context of an outbreak [14], and 4–20% in endemic settings [15], [16].

Compared with non-colonised patients, asymptomatic patients have higher rates of skin and environmental contamination (but less than that associated with symptomatic cases) [14], [17], [18], and *C. difficile* can be recovered from investigators’ hands after contact with colonized individuals [14]. Estimates of the extent of onward transmission within hospitals attributable to asymptomatic carriers vary considerably [19], [20]. In one study, nosocomial acquisition of a new *C. difficile* strain was preceded by initial introduction of the strain on to a ward by an asymptomatic carrier in 16/19 (84%) instances [19]. In another examining possible sources for recently diagnosed cases, 5/12 symptomatic contacts had a strain matching the new case compared to 1/19 asymptomatic contacts [20]. However these findings are based on relatively old studies and genotyping methods. Therefore, while onward transmission from asymptomatically colonised patients is clearly possible, its current relative importance as a source of CDI is unclear. We therefore prospectively screened a cohort of medical inpatients for asymptomatic carriage of *C. difficile*. Carriage isolates were then compared to all subsequent CDI cases in the same geographic region over the following 3 months for evidence of onward transmission from asymptomatic cases.

## Methods

### Setting and Participants

This study was performed at the Oxford University Hospitals NHS Trust, OUH. The OUH consists of 1600 beds across 4 hospital sites in the English county of Oxfordshire (population ∼600000). The hospitals provide all acute care to the population, >90% of all hospital services, and all diagnostic testing for *C. difficile* for hospital and community samples from the county.

All patients aged over 18 years admitted to a study ward were eligible for inclusion. The study was conducted on acute general medicine and geratology wards, as these specialities had the greatest historic CDI incidence. The choice of high prevalence wards was intended to provide the maximum number of CDI cases on the same ward as asymptomatic carriers, in order to investigate the potential for carrier to case transmissions. Patients diagnosed with CDI in the last 28 days, or with a current clinical suspicion of CDI (≥3 unformed stools in a 24 hour period) were excluded from this specific study (but samples/sequences from these cases were available for comparison with study participants, see below).

Recruitment was rotated around 8 study wards on a weekly to fortnightly basis (between 02 February 2012 and 10 June 2012) to reduce the nursing burden of sending stool samples from patients. Following informed consent (or the advice of an appropriate consultee in the case of patients without capacity) patients were recruited within 24 hours of ward admission where possible (however, no upper limit was set for the time between admission and enrolment).

### Samples

Patients were asked provide a sample of their first stool after enrolment, and then a sample at approximately 3 day intervals until discharge, and where possible a sample shortly prior to discharge.

Samples were cultured in weekly batches following the method of [21], which is very similar to that used in largest study of asymptomatic carriage to date [6]. Samples with colonies exhibiting a characteristic appearance, odour, and fluorescence consistent with *C. difficile* underwent whole genome sequencing as described in [5]. Briefly, indexed pools of 96 samples were sequenced using the Illumina HiSeq 2000 platform (Illumina Inc, San Diego, CA) generating 100 base pair paired-end reads. Reads were mapped with Stampy [22] to the *C. difficile* reference genome 630 (Genbank: AM180355.1) [23]. Samples were compared using single nucleotide variants (SNVs) identified with Samtools [24] after quality filtering. For each sequence the presence or absence of toxin genes and an *in silico* multilocus sequence type, MLST, was determined from *de novo* assemblies constructed with Velvet [25] using BLAST.

### CDI Cases for Comparison

All Oxfordshire hospital and community clinical CDI cases from April 2011 to August 2012 inclusive were considered for comparison. During this time all hospital inpatients with ≥3 unformed stools in 24 hours underwent *C. difficile* testing. Between April 2011 and March 2012 samples sent to the OUH microbiology laboratory underwent toxin EIA-testing, and EIA-positive samples were cultured. Between April 2012 and August 2012 samples were screened with a GDH test, with a follow up confirmatory EIA test in GDH-positive samples. All GDH-positive samples were cultured, regardless of the subsequent EIA result. This enabled potential *C. difficile* ‘excretors’ carrying *C. difficile*, but not currently producing toxin, to be included. Between April 2011 and August 2012 a total of 505 symptomatic *C. difficile* culture-positive samples were identified, 481 (95%) of which were successfully whole genome sequenced and available for comparison. A further 137 sequences were available from a *C. difficile* diagnostic study, conducted between April 2011 and September 2011, that were culture-positive, but EIA-negative [26].

### Epidemiological Data

Consent was obtained for use of hospital electronic data, including participant demographics (age, sex, postcode district (first 3–4 characters of the postcode)), hospital admission, discharge and ward movement data within the OUH, and previous and future routine *C. difficile* testing). Additional data on potential risks factors for *C. difficile* carriage were collected from patients using a questionnaire and hospital records (see **Table 1** and **Table 2**).

**Table 1:** Comparison of characteristics between participants returning and not returning at least one stool sample in 199 participants with questionnaire data.

Risk factor	Frequency in those not returning samples	Frequency in those returning samples	p value
Current diarrhoea, but not meeting CDI criteria	8/86 (9%)	22/111 (20%)	0.05
Nursing home, residential home, or institutional resident	8/87 (9%)	6/112 (5%)	0.40
Inflammatory bowel disease	0/86 (0%)	1/112 (1%)	1.00
Chronic kidney disease	17/86 (20%)	24/112 (21%)	0.86
Non-haematological malignancy	4/86 (5%)	17/112 (15%)	0.02
Haematological malignancy	2/86 (2%)	5/112 (4%)	0.70
Gastrointestinal surgery in current admission	1/86 (1%)	5/112 (4%)	0.24
Nasogastric tube in place	1/86 (1%)	3/112 (3%)	0.63
Overnight hospital stay in last 6 months	41/84 (49%)	43/111 (39%)	0.19
Outpatient hospital attendance in last 6 months	24/84 (29%)	33/111 (30%)	0.88
GP visits in last 6 months, n	87	112	0.30
0	4 (5%)	9 (8%)	
1–2	31 (36%)	36 (32%)	
3–9	28 (32%)	46 (41%)	
10+	24 (28%)	21 (19%)	
Visitor to hospital, nursing home, resident home	5/87 (6%)	3/111 (3%)	0.30
Work in healthcare	2/87 (2%)	3/111 (3%)	1.00
Healthcare worker in household	3/87 (3%)	9/111 (8%)	0.23
Previous *C. difficile* infection	0/87 (0%)	5/111 (5%)	0.07
Previous contact with a *C. difficile* infection case	3/87 (3%)	2/111 (2%)	0.66
Antibiotics in last 6 months	65/87 (75%)	90/112 (80%)	0.39
Steroids/Immunosuppressant in last 6 months	25/87 (29%)	28/112 (25%)	0.63
Gastric acid suppressant in last 6 months	25/87 (29%)	54/112 (48%)	0.006
Household pet	25/86 (29%)	27/111 (24%)	0.52
Physical contact with children ≥1 per month	49/86 (57%)	54/110 (49%)	0.31
Vegetarian diet	5/75 (6%)	7/111 (6%)	1.00
Foreign travel in last 6 months	3/87 (3%)	4/112 (4%)	1.00
Current smoker	10/87 (11%)	11/112 (10%)	0.82

Exact p values shown. Not all participants could answer all study questionnaire questions, as indicated in the dominator given in each line.

**Table 2:** Univariate risk factors for asymptomatic carriage of *C. difficile*.

Risk factor	*C. difficile* carriers(% total)	Non-carriers(% total)	Odds ratio, carriers:non-carriers	95% CI	p value
Current diarrhoea, but not meeting CDI criteria	5 (50%)	17 (17%)	4.94	(1.29, 19.0)	0.02
Nursing or residential home resident	2 (20%)	4 (4%)	6.13	(0.97, 38.7)	0.08
Chronic kidney disease	2 (20%)	22 (22%)	1.10	(0.21, 5.56)	0.90
Non-haematological malignancy	3 (30%)	14 (14%)	2.69	(0.62, 11.66)	0.21
Overnight hospital stay in last 6 months	6 (60%)	37 (36%)	2.59	(0.68, 9,79)	0.15
Time in hospital during current admission priorto enrolment, odds ratio per day*			1.00	(0.95, 1.05)	0.99
Outpatient hospital attendance in last 6 months	2 (20%)	31 (31%)	0.56	(0.11, 2.81)	0.47
GP visits in last 6 months					
0	2 (20%)	7 (7%)	1.77	(0.28, 11.1)	0.23
1–2	5 (50%)	31 (30%)	1		
3–9	2 (20%)	44 (43%)	0.28	(0.05, 1.55)	
10+	1 (10%)	20 (20%)	0.41	(0.03, 2.85)	
Healthcare worker in household	1 (10%)	8 (8%)	1.29	(0.14, 11.52)	0.82
Antibiotics in last 6 months	6 (60%)	84 (82%)	0.32	(0.08, 1.25)	0.12
Steroids/Immunosuppressant in last 6 months	5 (50%)	23 (23%)	3.43	(0.91, 12.9)	0.07
Gastric acid suppressant in last 6 months	7 (70%)	47 (46%)	2.73	(0.67, 11.2)	0.14
Household pet	3 (33%)	24 (24%)	1.62	(0.38, 7.00)	0.52
Physical contact with children ≥1 per month	3 (33%)	51 (51%)	0.49	(0.11, 2.07)	0.32
Current smoker	1 (10%)	10 (10%)	1.02	(0.11, 8.92)	0.98

There were no *C. difficile* carriers with the following risk factors: inflammatory bowel disease (present in 1/112 (1%) participants), haematological malignancy (5/112 (4%)), gastrointestinal surgery (5/112 (4%) with surgery in current admission, 12/112 (11%) with past surgery), current nasogastric tube placement (3/112 (3%)), previous CDI (5/112 (4%)), contact with a CDI case (2/112 (2%)), vegetarian diet (7/112 (6%)), and overseas travel (4/112 (4%)). *Given the study design, most participants spent relatively short amounts of time in hospital prior to enrolment, median (IQR) 2 (1–6) days.

### Analysis

Data obtained were used to estimate the point prevalence of asymptomatic carriage of *C. difficile*. Complete assessment of prevalence at admission and discharge, and therefore the incidence of acquisition, was not possible given the time delay between many participants’ recruitment and first bowel motion, and similarly relatively few participants providing more than one sample (see results and discussion).

Logistic regression was used to assess associations between the presence of *C. difficile* carriage in each participant’s first stool sample and risk factors for asymptomatic carriage. Univariate and multivariate models were fitted on cases with complete questionnaire data (n = 110/112 with any questionnaire data), with independent risk factors identified using backwards selection with the Akaike information criterion starting from a model including all variables with univariate p<0.20 [27], and then the final model re-fitted on all participants with complete data for the final variables included (n = 111). Because of the small size of the study we consider p<0.15 as marginal evidence of an effect and p<0.05 as significant evidence of an effect.

Whole genome sequences obtained from asymptomatic carriers were compared to CDI cases’ sequences obtained from 1 year prior to the study, during the study and for 3 months afterwards. Ward movements of cases and carriers sharing closely related *C. difficile* genomes were analysed for evidence of possible transmission events.

### Ethics

This study was approved by the Oxford C Research Ethics Committee (11/SC/0197). Samples from 20 asymptomatic study ward inpatients collected as part of routine infection control surveillance are also included in the analysis (no questionnaire data). Use of routinely collected samples and data, including from symptomatic patients, for the purposes of transmission analyses was approved by the Berkshire Research Ethics Committee (10/H0505/83) and National Information Governance Board (8–05(e)/2010) without requiring individual patient consent.

### Data Sharing

The sequences reported in this paper have been deposited in the European Nucleotide Archive Sequence Read Archive under study accession number <<<Details to be provided once accepted for publication>>> and are available at <<<Details to be provided once accepted for publication>>>.

## Results

### Participants and Samples

**Figure 1:**
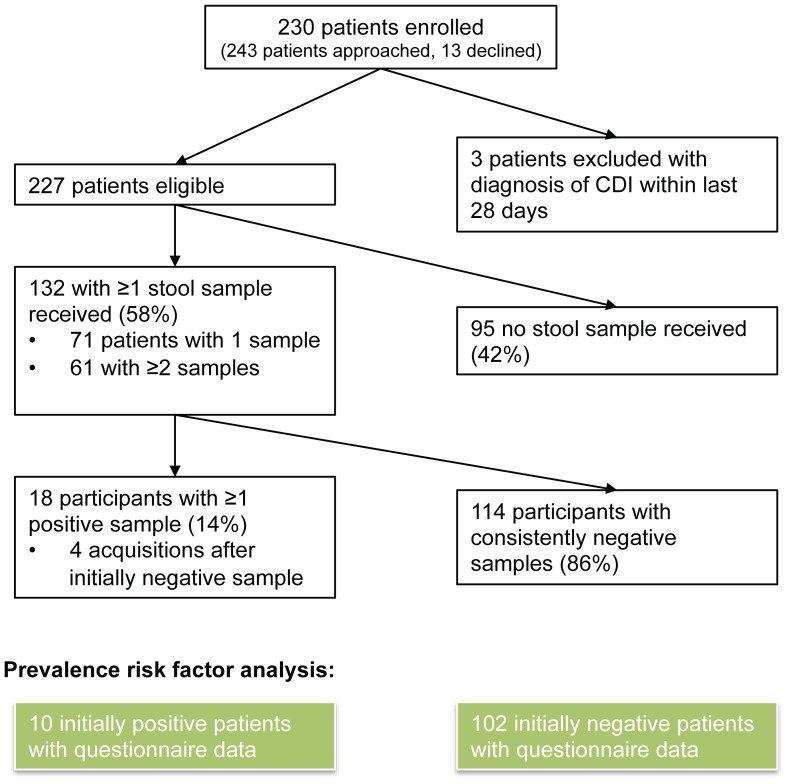
Asymptomatic study participants and samples.

**Figure 2:**
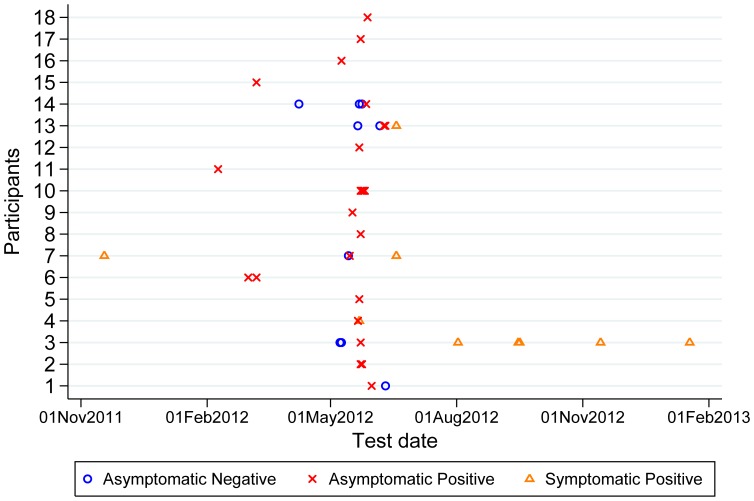
Temporal pattern of samples in participants with ≥1 positive sample. Asymptomatic positive and negative relate to samples obtained during the carriage study. Matching of study participants with the hospital admission and microbiology data allowed 4 participants with a subsequent symptomatic CDI positive samples to be identified, denoted symptomatic positive. 118 participants had consistently negative samples and are not plotted.

Two hundred and twenty-seven participants were recruited of 243 inpatients approached (13 declined to participate and a further 3 were ineligible due to a diagnosis of CDI in the last 28 days, Figure 1). A total of 296 stool samples were obtained from 132 participants (58% of recruited participants), 71 with a single sample and 61 with more than one sample, median (IQR) [range] samples per person 1 (1–2) [1]–[11]. Participants returning stool samples were similar to those not returning samples in age (median 83 versus 83 years, p = 0.96), and putative risk factors for carriage, with the exceptions that they were more likely to have current diarrhoea not meeting CDI criteria (i.e. patient reported loose/frequent stool, but <3 stool samples in 24 hours taking the shape of their container) (p = 0.05), have a non-haematological malignancy (p = 0.02), and have taken gastric acid suppressant medication in the last 6 months (p = 0.006; side effects of proton pump inhibitors and H_2_-receptor antagonists include diarrhoea, and therefore may have increased the rate of sample return in this group) (**Table 1**). A total of 28 samples from 18 participants tested positive for *C. difficile* (14% of those tested). 14 participants (11%) tested positive on their first sample, with a further 4 participants (3%) testing positive after 1, 2, 2, and 3 initially negative samples (Figure 2). Five of the 14 participants positive on their first sample provided subsequent samples; Participant 1 had the only apparent loss of carriage with a positive sample followed by a negative sample, in contrast the 4 other participants were persistently positive on multiple testing (Figure 2). The median (IQR) [range] time from study ward admission to enrolment was 2 (1–5) [0–44] days; patients were admitted via a separate admissions unit, such that an additional median (IQR) [range] 1 (0–2) [0–56] days elapsed in hospital before admission to a study ward.

### Relationship between Carriage and Previous/Subsequent Disease

Participants were linked with hospital admission and microbiology data to identify those previously or subsequently testing positive for *C. difficile* as a result of clinical testing in symptomatic patients (Figure 2). Four such participants were identified. Participants 4, 13 and 7 had a routine microbiology laboratory diagnosis of CDI 1, 8 and 35 days respectively after a positive carriage test in the study. In all 3 cases no SNVs were found between the asymptomatic study sample and the subsequent symptomatic positive sample. Participant 7 also had a positive routine laboratory CDI sample from 178 days prior to enrolment which was also genetically indistinguishable to the participant’s later samples, suggesting the single negative sample obtained from this participant may have been falsely negative, or that this strain was archived undetectably within the participant’s intestinal flora and selected for by antibiotics received in hospital. In contrast, participant 3 tested positive 86 days following a positive asymptomatic study sample, but with a different infecting *C. difficile* lineage, 11892 SNVs different from the carriage isolate. Participants 3 and 4 reported loose or more frequent stool at the time of enrolment, but less than the ≥3 unformed stools in 24 hours required to meet criteria for routine *C. difficile* testing. Participants 7 and 13 had no diarrhoeal symptoms at enrolment.

### Risk Factors for Carriage

Questionnaire data obtained at participant enrolment were used to assess risk factors for *C. difficile* carriage in participants’ first stool sample. A total of 112 (85%) of the 132 participants with ≥1 stool samples were able to complete the study questionnaire. Ten participants initially tested positive, a 9% point prevalence for asymptomatic *C. difficile* carriage in the population tested.

Univariate odds ratios for the risk factors assessed are presented in **Table 2**. The risk factor for carriage with the strongest statistical evidence was current diarrhoea not meeting diagnostic criteria for CDI. Patients with ≥3 unformed stools in a 24 hour period, i.e. meeting OUH criteria for CDI testing, were excluded from the study. However, 22 participants reported loose or more frequent stool than usual, but did not meet the local criteria for CDI testing. It is therefore possible that the five of these 22 patients testing positive for *C. difficile* actually should have had a diagnosis of mild CDI (none died within the 30 days following their sample despite not receiving CDI treatment). Non-significant univariate trends towards increased risk of *C. difficile* carriage (p<0.15) were seen with nursing or residential home residence, an overnight stay in hospital in the last six months, steroids or another immunosuppressant in the last six months, and gastric acid suppressant medication in the last six months. Intriguingly any antibiotic exposure in the last six months had a trend towards lower risk of *C. difficile* carriage.

Independent risk factors in a multivariate model are shown in **Table 3** and include current diarrhoea, but not meeting CDI criteria, overnight hospital stay in the last six months and steroids or another immunosuppressant in the last six months. As in the univariate analysis, antibiotics in the last six months was associated with lower odds of *C. difficile* carriage. The lower odds of *C. difficile* carriage with prior antibiotics in the multivariate model is particularly driven by the low number of carriers in the group of participants receiving antibiotics without hospital exposure in the last six months (1/51, 2%, **Table 4**). However, as illustrated, the small number of carriers means that a single change in the number of carriers could have changed odds ratio estimates substantially. The timing of antibiotic exposure in participants and the antibiotics taken is given in **Table 5**. 44 (39%) participants were taking antibiotics at enrolment and a further 14 (12%) had taken antibiotics in the 2 weeks prior to enrolment. Co-amoxiclav was the most commonly used antibiotic in 39 (35%) participants.

**Table 3:** Multivariate risk factors for asymptomatic carriage of *C. difficile.*

	Participants with questionnaire data			Excluding participants reporting mild diarrhoea		
Risk factor	Odds ratio	95% CI	p value	Odds ratio	95% CI	p value
Current diarrhoea, but not meeting CDI criteria	10.0	(1.96, 50.9)	0.006	–		
Overnight hospital stay in last 6 months	5.53	(1.06, 28.8)	0.02	5.19	(0.58, 46.0)	0.14
Antibiotics in last 6 months	0.07	(0.01, 0.45)	0.005	0.04	(0.002, 0.51)	0.01
Steroids/Immunosuppressant in last 6 months	7.19	(1.32, 39.1)	0.02	16.5	(1.25, 215)	0.03

The left hand side of the table includes all participants with questionnaire data, the right hand side excludes 22 patients with loose or more frequent stool, but not meeting CDI diagnosis criteria (5 carriers and 17 non-carriers). No significant pairwise interactions were identified, but given the relatively small sample size the power to detect these is low. Excluding one participant with missing hospital exposure data.

**Table 4:** Relationship between overnight hospital stays and antibiotic exposure in the last six months amongst *C. difficile* carriers and non-carriers.

Risk factor combination	*C. difficile* carriers (% row total)	Non-carriers (% row total)
Hospital + Antibiotics	5 (13%)	34 (87%)
Overnight hospital only	1 (25%)	3 (75%)
Antibiotics only	1 (2%)	50 (98%)
Neither	3 (18%)	14 (82%)

111 of the 112 participants with questionnaire data completed both questions on hospital exposure and antibiotic use.

**Table 5:** Type and duration of antibiotic exposure at study enrolment in *C. difficile* carriers and non-carriers.

	Initial *C. difficile* culture			
Antibiotic exposure inlast 6 months	Negative	Positive	Total	
**Total participants**	102	10	112	
**No Antibiotics**	18	4	22	
**One or more antibiotics**	84	6	90	
**Amoxicillin**	4		4	
Current at enrolment	2			
≤2 weeks previously	1			
>2 weeks ago	1			
**Cephalosporin**	3		3	
Current at enrolment	3			
**Co-amoxiclav**	21	3	24	
Current at enrolment	13			
≤2 weeks previously	8	3		
**Flucloxacillin**	4		4	
Current at enrolment	4			
**Macrolide**	2		2	
Current at enrolment	1			
≥2 weeks ago	1			
**Trimethoprim**	2		2	
Current at enrolment	1			
≤2 weeks previously	1			
**Other**	1		1	
>2 weeks ago	1			
**Multiple** *	20	2	22	
Current at enrolment	18	2		
>2 weeks ago	2			
**Unknown Agent**	27	1	28	
<2 weeks previously	1			
≥2 weeks ago	3			
Unknown time(but <6 months)	23	1		

* Of those taking multiple agents, 15 participants’ regimes included co-amoxiclav, 7 participants a macrolide, and 3 a cephalosporin.

In a sensitivity analysis, 22 participants reporting loose or more frequent stool, but not meeting CDI diagnosis criteria, were excluded. Of the remaining participants 5/90 (6%) were *C. difficile* carriers. The same risk factors remained in the multivariate model, with broadly similar point estimates for the odds ratios, however with greater uncertainty around these estimates given the smaller numbers of participants in the analysis (**Table 3**).

### Genetic Relationships between Carriage and Symptomatic Isolates

The first positive isolate from the 18 participants with ≥1 positive sample(s) underwent whole genome sequencing. Positive samples were obtained between 09 February 2012 and 09 June 2012 (1 in February, 2 in March, 14 in May, and 1 in June 2012, the variation reflecting changes in recruitment and sampling intensity). These sequences were compared with sequences obtained from all Oxfordshire hospital and community symptomatic *C. difficile* positive samples processed by the routine OUH microbiology laboratory between April 2011 and August 2012. All samples were mapped to the CD630 reference genome, with a mean of 85.1% of the reference genome called in the 18 carriage samples, and 84.3% across the 618 comparison samples. Mean mapped read depths were 88.9 and 84.3 respectively.

Thirteen of the asymptomatic carriage isolates sequenced (72%) were toxigenic and from disease-causing lineages (**Table 6**). Sequence types, STs, obtained from symptomatic cases and toxigenic strains from asymptomatic carriers overlapped (**Table 7**). No asymptomatic isolates from the epidemic ST1 lineage were seen, but this is consistent with ST1 (ribotype 027/NAP1) having largely ceased to be a cause of symptomatic disease in Oxfordshire since 2009 [5], affecting only 9 CDI cases in our study period. Interestingly asymptomatic carriage of an ST11 (ribotype 078), an emerging hypervirulent strain from clade 5, was seen in one participant, with 27 symptomatic cases over the study period.

**Table 6:** Asymptomatic carriage isolates: multilocus sequence types, clades and toxin status.

ST	Clade	Toxin A/B	Frequency	Participant Id
2	1	+/+	2	1, 16
3	1	+/+	2	14, 18
6	1	+/+	2	6, 10
7	1	−/−	1	15
11	5	+/+	1	9
13	1	+/+	1	17
18	1	+/+	1	13
26	1	−/−	1	8
29	1	−/−	2	2, 3
35	1	+/+	2	7, 11
46	1	+/+	1	5
49	1	+/+	1	4
125	1	−/−	1	12

Participant id follows that used in Figure 2.

**Table 7:** Comparison of multilocus sequence types (STs) observed in symptomatic cases and asymptomatic participants, 01 April 2011 to 31 August 2012.

ST	Asymptomatic participants (% total)n = 18		Routinely diagnosed CDI (% total)n = 481		Toxin EIA-negative, culture positive (% total)n = 137		All symptomatic comparison samples (% total)n = 618	
2	2	(11%)	42	(9%)	14	(10%)	56	(9%)
6	2	(11%)	47	(10%)	6	(4%)	53	(9%)
8			43	(9%)	10	(7%)	53	(9%)
10			36	(7%)	6	(4%)	42	(7%)
44			30	(6%)	9	(7%)	39	(6%)
14			21	(4%)	8	(6%)	29	(5%)
7	1 (−)	(6%)	14	(3%)	14	(10%)	28	(5%)
15			16	(3%)	12	(9%)	28	(5%)
11	1	(6%)	24	(5%)	3	(2%)	27	(4%)
5			18	(4%)	3	(2%)	21	(3%)
3	2	(11%)	15	(3%)	2	(1%)	17	(3%)
26	1 (−)	(6%)	8	(2%)	7	(5%)	15	(2%)
58			14	(3%)	1	(0.7%)	15	(2%)
9			11	(2%)	2	(1%)	13	(2%)
54			13	(3%)	0	(0%)	13	(2%)
16			11	(2%)	1	(0.7%)	12	(2%)
35	2	(11%)	8	(2%)	4	(3%)	12	(2%)
46	1	(6%)	11	(2%)	0	(0%)	11	(2%)
37			8	(2%)	2	(1%)	10	(2%)
1			7	(1%)	2	(1%)	9	(1%)
49	1	(6%)	8	(2%)	1	(0.7%)	9	(1%)
12			4	(0.8%)	4	(3%)	8	(1%)
13	1	(6%)	6	(1%)	2	(1%)	8	(1%)
17			4	(0.8%)	3	(2%)	7	(1%)
55			4	(0.8%)	2	(1%)	6	(1%)
22			5	(1%)	0	(0%)	5	(0.8%)
33			4	(0.8%)	1	(0.7%)	5	(0.8%)
36			4	(0.8%)	1	(0.7%)	5	(0.8%)
4			2	(0.4%)	2	(1%)	4	(0.6%)
43			3	(0.6%)	1	(0.7%)	4	(0.6%)
45			3	(0.6%)	1	(0.7%)	4	(0.6%)
48			1	(0.2%)	3	(2%)	4	(0.6%)
29	2 (−)	(11%)	2	(0.4%)	1	(0.7%)	3	(0.5%)
42			3	(0.6%)	0	(0%)	3	(0.5%)
18	1	(6%)	1	(0.2%)	1	(0.7%)	2	(0.3%)
56			2	(0.4%)	0	(0%)	2	(0.3%)
107			1	(0.2%)	1	(0.7%)	2	(0.3%)
122			2	(0.4%)	0	(0%)	2	(0.3%)
125	1 (−)	(6%)	1	(0.2%)	1	(0.7%)	2	(0.3%)
205			0	(0.0%)	2	(1%)	2	(0.3%)

Asymptomatic carriers without the toxin A and B genes are indicated by “(−)”, see Table 6. Symptomatic cases are broken down by whether the case was diagnosed via the routine laboratory or as part of a *C. difficile* diagnostic study (toxin EIA-negative, culture positive) (see Methods). Single isolates were obtained from symptomatic cases from the following STs: 34, 51, 53, 66, 67, 75, 90, 103, 124, 133, 139, 150. In addition 10 symptomatic samples were obtained from 9 novel STs. In 6 symptomatic samples it was not possible to recover a complete *in silico* ST.

**Figure 3:**
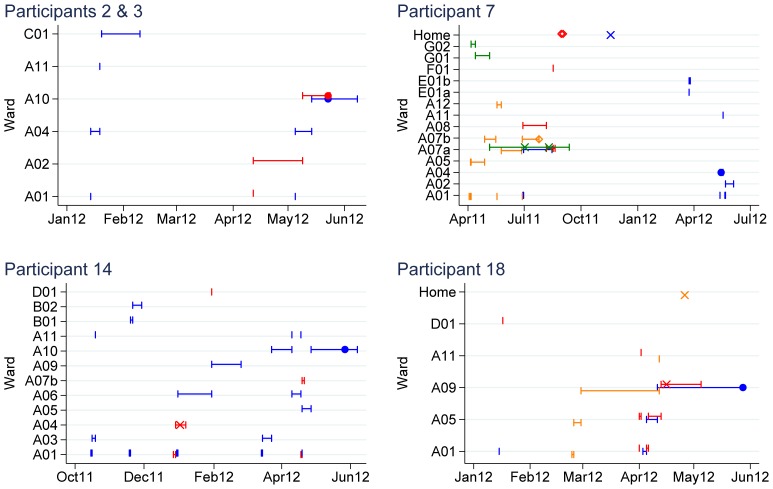
Epidemiological relationships between 4 asymptomatic study participants and genetically related cases. Study participants are shown in blue, with the exception of participant 3, shown in red in the first panel. Symptomatic cases are shown as different colours, and are distinct across different panels. Positive asymptomatic samples from study participants are shown as filled circles. Positive symptomatic samples are shown as crosses. EIA-negative culture-positive samples are shown as diamonds. Ward stays are shown as horizontal lines with capped ends. Wards sharing the same specialty and hospital share the same initial letter; adjacent wards forming a single unit have the same letter and number and are followed by a lower case letter.

The number of SNVs between each of the carriage samples and the closest prior and subsequent symptomatic or asymptomatic sample is shown in **Table 8**. In keeping with the significant diversity observed in symptomatic patients [5], 9/18 (50%) carriage isolates were >10 SNVs different from all other carriage or disease samples. However, several isolates fell within the diversity observed in common disease-causing sequence types. Five participants had a sample that was ≤2 SNVs from at least one other symptomatic or asymptomatic sample (i.e. consistent with transmission based on rates of *C. difficile* within host diversity and evolution [5]). Participants 2 and 3 had non-toxigenic samples within 1 SNV of each other, but were ≥30 SNVs different from any symptomatic case. Participants 7, 18, and 14 had samples which were ≤1 SNV from at least one previous symptomatic toxin A/B positive sample. Epidemiological relationships between CDI cases and asymptomatic participants with all samples genetically related within ≤2 SNVs are shown in Figure 3.

**Table 8:** Single nucleotide variants, SNVs, between 18 asymptomatic carriage samples and most closely genetically related prior and subsequent symptomatic/asymptomatic sample.

Participant	ST	Toxin A/B	SNVs to most closely related sample	SNVs to most closely related prior sample	SNVs to most closely related subsequent sample
7* §	35	+/+	0	0	21
2	29	−/−	1	1	4840
18	3	+/+	1	1	13
3* †	29	−/−	1	1	4817
14*	3	+/+	1	1	278
10	6	+/+	5	5	21
11	35	+/+	6	10	6
6	6	+/+	8	14	8
1	2	+/+	10	10	14
16	2	+/+	18	21	18
8	26	−/−	21	21	37
5	46	+/+	24	24	48
17	13	+/+	25	37	25
15	7	−/−	57	4558	57
12	125	−/−	61	3006	61
9	11	+/+	461	461	1294
4§	49	+/+	944	1627	944
13* §	18	+/+	1656	2299	1656

Participants are ordered by the number of SNVs to the most closely related sample. The participant numbering follows the same scheme used in Figure 2.

* indicates acquisition following an initially negative sample.

§ indicates subsequently developed disease with the same strain,

† indicates subsequently developed disease with a different strain.

Several plausible transmission events involving asymptomatic *C. difficile* carriers as recipients can be seen from the combined genetic and epidemiological data. Participants 2 and 3 shared time on a ward together before both were found to be asymptomatic carriers with isolates 1 SNV different, either representing transmission from one asymptomatic participant to another, or both having been exposed to an un-assayed common source. Participant 18 shared time on a ward with a symptomatic patient before being found to carry *C. difficile* only 1 SNV different to the symptomatic case. The epidemiological linkage between participant 14 and the most closely genetically related case is less strong, with only a short overlap between the two patients 6 months prior to the participant’s sample. Participant 7′s asymptomatic carriage strain appears to have been acquired nearly a year previously, with an intervening symptomatic episode, while sharing time on a ward with 3 other cases, 2 with identical sequences, and 1 a single SNV different. Interestingly 2 of these 3 cases were EIA-negative on an initial screen, although toxin genes were present in the sequences. Although it can be difficult to infer the direction of transmission with often only a single sample from asymptomatic carriers and symptomatic cases, in this relatively small study, no clear evidence of onward transmission from an asymptomatic case was seen despite sequencing nearly all of the subsequent CDI cases for 3 months.

## Discussion

In this study we determine the prevalence of asymptomatic *C. difficile* carriage in medical inpatients in an endemic setting with limited case-to-case transmission [5], define risk factors for carriage, and investigate its role in onward transmission. The estimated carriage point prevalence of 6% or 9% (in participants with questionnaire data, depending on the definitions used to define “asymptomatic”) is consistent with previous estimates in other populations [6], [12]. The majority of asymptomatically carried strains were toxigenic and from disease-causing genotypes.

Previous hospital exposure and steroid/immunosuppressant medication were identified as independent risk factors for carriage supporting recently published data from a similar study of 320 patients in North America [28]. Patient reported loose or increased stool frequency not meeting the threshold for CDI testing (≥3 unformed stools in 24 hours) also increased the risk of carriage; arguably these patients may have mild CDI not captured by the commonly used threshold for testing in place in our institution. Mandatory CDI reporting [29] may act as a disincentive to diagnosing such cases, as may the financial penalties imposed for excess cases in the UK [30]. Although the benefit of treating mild CDI is uncertain [31], these patients are still a potential source of onward transmission and therefore overly restrictive diagnostic definitions may in fact hinder infection control.

The finding that patient-reported antibiotic use in the last six months was associated with decreased odds of carriage was unexpected. Other studies have found no association with antibiotic use [6], [28] or that antibiotics were associated with increased carriage rates [32]. There are several possible explanations for our finding. Enrolment to the study was conditional on participants not having had a recent CDI; it is therefore possible that a proportion of patients with *C. difficile* carriage receiving antibiotics were selected out, having already developed CDI. Recall bias is a potential concern regarding antibiotic exposure prior to the current hospital admission (hospital records were used to determine antibiotic exposure during the current hospital admission), particularly as our study depended on next of kin recall in participants without capacity. More active participants with less healthcare exposure are possibly more likely to recall antibiotic use and have lower intrinsic risk of carriage. *C. difficile* strains sensitive to antibiotics taken by participants may also have been suppressed or removed by antibiotic use; many participants were taking antibiotics at enrolment or had been exposed in the previous 2 weeks. Trends towards gastric acid suppression and nursing/residential home residence increasing carriage were seen on univariate analysis and may merit further investigation in larger studies in future.

By using whole genome sequencing combined with ward movement data several potential acquisitions of asymptomatic carriage strains from symptomatic cases were observed. However, none of the 13 asymptomatically carried toxigenic isolates were involved in onward transmission to any CDI case diagnosed in the region within ≥3 months, i.e. within current consensus on common incubation periods [3], [33]. This suggests onward transmission from asymptomatic patient carriers may be infrequent (one-tailed 97.5% exact confidence interval for proportion of colonisations leading to a secondary case 0, 0.25). Perhaps this is unsurprising given asymptomatic carriage is much more prevalent than disease. If, based on this and previous studies, ∼5% of those admitted to hospital are colonised, onward transmission per case could be infrequent but still contribute substantially to the overall burden of disease. For example, if rates of CDI range from 1 to 10 per 1000 admissions [34], then as few as 1 in 10 to 1 in 100 asymptomatic carriers respectively would need to transmit for transmission from asymptomatic carriage to account for 50% of all cases. Such a pattern of transmission would be consistent with the considerable genetic diversity seen in disease causing strains [5]. A considerably larger study than this would be needed to estimate more precisely the proportion of asymptomatic carriers transmitting onwards to a new CDI case. For example, if the expected proportion of asymptomatic carriers transmitting onward to a case is 5%, then 298 carriers (and all associated cases) would be needed for the upper limit of the 95% confidence interval around the estimated proportion of asymptomatic carriers transmitting to be <10% with 0.95 probability [35]. Even with asymptomatic carriage rates of ∼10% this would involve a study with ∼3000 participants.

This scenario has implications for further research and for control. Research studies investigating the role of asymptomatic carriers either need to prospectively identify and recruit considerable numbers of carriers to detect a small proportion involved in onward transmission, or studies need to rapidly follow up the potential sources of each new case immediately after diagnosis before the new cases have a chance to infect their contacts themselves (as done in the study of Samore *et al* [20]).

If a low transmission rate from a substantial minority of asymptomatic carriers nevertheless is an important source of disease, then this has substantial implications for hospital infection control, as screening and isolation or *C. difficile* eradication in these carriers may interrupt transmission. However, the number needed to isolate/treat to prevent one transmission is likely to be large, albeit possibly still cost-effective if the prevalence of carriage and probability of onward transmission is high enough [36] or screening can be targeted to those most likely to carry [28]. In a small study, vancomycin treatment did not reduce long-term carriage, but did transiently render subjects culture-negative during and immediately after treatment [37]. Transient suppression of carriage would probably be sufficient to reduce risk of onward transmission throughout most patients’ hospital admission as *C. difficile* was not detected until 20±8 days after the end of a 10 day vancomycin course. However there is no direct evidence that treating asymptomatic carriers or instituting more stringent isolation of carriers leads to less transmission [38]. The closest to this kind of evidence is a study in a Belgian leukaemia unit, where a combination of environmental cleaning and renovation together with treatment of asymptomatic carriers with vancomycin reduced CDI rates markedly [39].

A major challenge in conducting this study was obtaining samples, and especially serial samples, from patients. As designed, opportunistically sampling patients when they open their bowels, the study was highly acceptable to patients (and the relatives of patients without capacity) with 230/243 (95%) of those approached agreeing to participate. However obtaining samples therefore depended on the goodwill of ward nursing staff to assist patients in returning samples, and on participants’ bowel habit. Both factors contributed to relatively low rates of sample return, with only just over half of participants recruited returning one or more samples. An alternative approach would have been to take rectal or perianal swabs from patients. As this is more invasive, participation rates are likely to be lower; in a large Canadian study using rectal swabs in some participants (and also obtaining blood samples) participation rates were 57% [6]. However this approach enables samples to be obtained by dedicated research staff, and provides control over the timing of samples from consenting patients. Although overall either approach seems to result in similar proportions of patients being sampled at least once, if sufficient resources are available, direct swabbing enables much more complete serial sampling of participants. Sampling by dedicated research staff is also likely to ensure sample return is more sustainable.

Another important limitation in any carriage study is how closely the limit of detection of the organism corresponds to complete absence of the organism. In this study the culture methods used are well supported and follow the largest study of asymptomatic colonisation to date [6], [40]. Only a single possible false negative result was observed in a patient positive the following day. Undetected carriage may also contribute to transmission; although it seems plausible that *C. difficile* carried in detectable quantities is more likely to be transmitted, and therefore likely to be a more significant potential source of infection than undetectable carriage.

Overall, this study supports data from other settings on the prevalence of asymptomatic carriage, and provides clear evidence for carriage of disease-causing strains. It also suggests potential patient populations that may be at increased risk of carriage. Whole genome sequencing provides a powerful tool for studying potential transmission events involving asymptomatic carriers. Preliminary findings here do not suggest these transmission events are common at the level of individual carriers, but rather transmission may be a relatively rare event. However as asymptomatic carriers are a relatively large pool of patients, they may still have an important role in onward transmission leading to disease, which could be quantified in larger studies.
